# Effect of different *Lactobacillus* species on volatile and nonvolatile flavor compounds in juices fermentation

**DOI:** 10.1002/fsn3.1010

**Published:** 2019-06-20

**Authors:** Shumao Cui, Nan Zhao, Wenwei Lu, Fang Zhao, Songyou Zheng, Weijun Wang, Wei Chen

**Affiliations:** ^1^ State Key Laboratory of Food Science and Technology Jiangnan University Wuxi China; ^2^ School of Food Science and Technology Jiangnan University Wuxi China; ^3^ Institute of Agro‐Products Processing Science and Technology Sichuan Academy of Agriculture Sciences Chengdu China; ^4^ Zhejiang Liziyuan Food Co., Ltd Jinhua China; ^5^ National Engineering Research Center for Functional Food Jiangnan University Wuxi China; ^6^ Beijing Innovation Centre of Food Nutrition and Human Health Beijing Technology and Business University (BTBU) Beijing China

**Keywords:** flavor, GC‐MS, juices fermentation, *Lactobacillus*, taste

## Abstract

*Lactobacillus* is the dominant genus during fruit and vegetable juices (FVFs) fermentation, which are the key factors for taste and flavor. This study was performed to investigate the effects of different *Lactobacillus* spp. on profile of volatile flavor compounds and nonvolatile taste compounds in FVFs fermentation. A total of 14 compounds were identified as discriminant flavor and taste markers for fermented FVFs via gas chromatography–mass spectrometry (GC‐MS)‐based multimarker profiling. The PCA score plot and PLS‐DA showed that different FVFs were divided into three distinct types, suggesting that the different species significantly affect the volatile and nonvolatile compounds profiles of FVFs. *Lactobacillus casei *and *Lactobacillus rhamnosus* (Type A FVFs) might make a greater contribution to the umami taste. *Lactobacillus plantarum* and *Lactobacillus acidophilus* (Type B FVFs) make a greater contribution to the sour taste. *Lactobacillus fermentum *may be an potential critical contributor to produce volatile compounds. We reveal that different *Lactobacillus *strains play different roles in modifying these compounds related to flavor and taste features.

## 1 INTRODUCTION

Fresh fruits and vegetables are strongly recommended in the human diet on basis of their abundant antioxidant, vitamins, dietary fibers, and minerals. Nevertheless, great majity of fresh vegetables and fruits have a very sht shelf life due to rapid bacterial spoilage. Among the various food processed options, fermentation may be regarded as an effective and simple biotechnology fmaintainingimproving the safety, sensy, nutritional, and shelf‐life properties of fruits and vegetables.

Lactic acid bacteria (LAB) has frequently employed in the fermented vegetables such as cucumbers, cabbages, and olives fermentation (Rodríguez et al., 2009). Recently, LABs were also successfully used to ferment vegetables and fruits (FVFs) such as carrots, French beans and marrows (Di Cagno et al., 2008), red and yellow peppers (Cagno, Surico, Minervini, et al., 2009), tomato juices (Cagno, Surico, Paradiso, et al., 2009), and pineapple fruits (Di Cagno et al., 2010). Among these LABs, *Lactobacillus* is the most dominant genus in the vegetable and juice fermentation. Various species of *Lactobacillus* were isolated from different kind of FVFs, including *Lactobacillus fermentum *(*L. fermentum*), *Lactobacillus plantarum* (*L. plantarum*), and *Lactobacillus casei* (*L. casei*). According to the past studies, different species of *Lactobacillus* have diverse metabolic responses during the fermentation and have different effects on the flavor of FVFs. Although diverse species of *Lactobacillus* was isolated from fermented vegetable and fruit juices, less attention is paid to the effect of metabolic responses from different species of *Lactobacillus* on the flavor of FVFs. Thus, which species of *Lactobacillus* is more suitable for producing high flavor quality vegetables and fruits fermentation production remain unclear.

Metabolomics is an avenue applied to quantify the global metabolites in biological samples (Dunn, Bailey, & Johnson, 2005; Patti, Yanes, & Siuzdak, 2012). Nowadays, large amount of researchers have used metabolomics to discover the biomarker metabolites generated in biotechnological food systems, which has contribution to the quality of food (Baek et al., 2010; Kang et al., 2011; Kang, Lu, & Zhang, 2015; Kang, Zhu, Lu, & Zhang, 2015). In past studies, targeted flavor metabolites (sugars and organic acids) have been used to estimate the contribution of *Lactobacillus* in FVFs, but these compounds might be limited to a synthetic and global representation of the flavors and nutritional chemicals in FVFs. Hence, an untargeted metabolomics analysis is used in the present study to compensate for the limitations of the aforementioned targeted methods when evaluating the contribution of *Lactobacillus* in FVFs.

The major objective of this research was to clear the marker metabolites generated by different species of *Lactobacillus*, which is contributed to the flavor of FVFs and reveal the roles of diverse species of *Lactobacillus* in formation of flavor compounds in FVFs.

## 2 MATERIALS AND METHODS

### 2.1 Bacterial strains and culture conditions

To investigate the species‐dependent characteristics of FVFs, *L. plantarum* CCFM 1, *L. casei* CCFM 2, *Lactobacillus acidophilus* CCFM 8, *L. fermentum* CCFM 19, and *Lactobacillus rhamnosus* CCFM 24 were selected to make FVFs. The five strains were isolated from Paocai (a Chinese traditional fermented vegetables) and obtained from Culture Collection of Food Microorganisms of Jiangnan University (Wuxi, China). Colonies were isolated from MRS (De Man, Rogosa, & Sharpe, 1960) agar and grown in MRS broth to achieve activation. The preculture was prepared at 37°C for 18 hr. The bacterial suspensions were used as inocula for the vegetable and fruit juice fermentation.

### 2.2 Preparation of fermented vegetable juices

Five kinds of fruit and vegetable juice obtained by squeezing fresh fruits and vegetables were filtered with filter paper, and then centrifuged for 20 min with 10,000 *g*. The supernatants were used to make mixed vegetable and fruit juice, which include apple, carrot, tomato, cucumber, and haw (40:25:15:15:5). The mixed juice was sterilized for 20 min at 80°C. Five strains were inoculated (2%, v/v) into mixed vegetable and fruit juice, respectively, to prepare the FVFs. The inoculated juices were fermented at 30°C for 24 hr and stored at 4°C for another 3 days. To identify the discriminant compounds among different samples, FVFs were sampled at 48 hr. To reveal the roles of *Lactobacillus* on these markers, FVFs were sampled at 0, 6, 12, 24, and 48 hr. Three biological replicates of the fermented samples were collected for the subsequent GC/MS detection.

### 2.3 Gas chromatography–mass spectrometry (GC‐MS) analysis

#### 2.3.1 Nonvolatile compounds

Firstly, as for GC‐MS analysis, the supernatant of the juice was divided into 50 ml, and an aliquot of 50 μl of the supernatant was transferred into a tube. Ribitol (15 μl of a 0.20 mg/ml solution) was added as an internal standard. Subsequently, the samples were dried in order to further derivatization referring to the methods of Zhao et al. (2016). Then transferring the sample into GC vials for further analysis, samples (1 µl) were injected into the column under the mode of separation. The samples were analyzed by GC (GC‐2010 Plus, Shimadzu, Japan) fitted with quadrupole MS (GCMS‐QP2010 Ultra, Shimadzu, Japan) using a Rtx‐5MS capillary column (30 m, 0.25 mm inner diameter, and 0.25 μm thickness). The parameter of injection temperature and the transfer line and the ion source were 240, 220, and 220°C, respectively. Helium was usually used as gas of carrier, flowing at a stationary linear speed of 35.0 cm/s. The temperature of oven was increased from 70 to 230°C at a speed of 5°C/min. Subsequently, the temperature was increased to 320°C at a speed of 90°C/min and then keeping for 5 min. The scanning range was from 33 to 600.

#### 2.3.2 Volatile compounds

The volatile compounds in different samples were extracted by headspace solid‐phase microextraction (HS‐SPME). The procedure of sample extraction and testing was referred to Zhao et al with some modifications making. Before the analysis, 5 ml FVFs holding in 20°C for 10 min. Methyl heptanoate (20 mg/ml; Sigma‐Aldrich Milwaukee, WI) that was added into each sample served as the internal standard in order to insure the retention time of the peaks of volatile component. DVB/CAR/PDMS fibers (Supelco, Bellefonte, PA, USA) and aoc‐5000 Plus are often used in automatic injection (Shimadzu, Japan). After balancing 30 min under 50°C in oscillator, the fiber was placed in the headspace under 50°C for 30 min. It takes about 2 min to desorption in splitless mode at 240°C. We used a GC (GC‐2010 plus, Shimadzu, Japan) equipped with four‐pole MS (GCMS‐QP2010 Ultra, Shimadzu, Japan) using an Rtx‐wax capillary column (30 m, 0.25 mm ID, 0.25 mm thickness). The temperature of injection and the transfer line and the ion source were 240, 220, and 220°C, respectively. Helium is often used as carrier gas to assist sample flow at a constant linear speed of 35 cm/s. Temperature program is divided into three phases: the first stage is that holding 3 min at 40°C, then increasing the temperature from 40 to 130°C at a line speed of 5°C/min and holding for 5 min at 130°C; the second stage is that increasing temperature from 130 to 155°C at a linear speed of 25°C/min; and the third stage is that increasing the temperature from 155 to 220°C at a linear speed of 5°C/min and holding 5 min at 220°C. Three parallel experiments are required for all samples. The identification of VOCs can be determined by comparing with NIST 2011 standard mass spectrometry database, according to the retention time and similarity matching degree of mass spectrometry over 85% (Wu et al., 2015; Zhao, Tang, & Ding, 2007).

### 2.4 Spectral data processing

XCMS estimates the MS online refer to the preceding introduction (Zhao et al., 2016). In order to implement MS response drift correction during its operation, manual normalization of volatile and nonvolatile compounds spectra was performed by regulating the peak intensity for heptanoic acid methyl ester and ribitol internal standard, respectively.

### 2.5 Metabolite identification

Metabolites were identified by comparison with the standard mass spectrometry database (NIST 2011). Retention time is often used as a qualitative indicat, and the potential metabolites were identified by the degree (me than 90%) of similarity with mass spectra. All putative markers were affirmed by reference substance.

### 2.6 Statistical analysis

Principal component analysis (PCA) and partial least squares discriminant analysis (PLS‐DA) were performed using SIMCA‐P 12.0 (Umetrics AB, Umea, Sweden). To validate models, a sevenfold validation was applied to the PLS‐DA model and the reliabilities of the models were further rigorously validated by a permutation test (*n* = 100). Student's *t* test and Duncan's multiple range test were performed using SPSS 16.0 (SPSS Inc., Chicago, IL) to determine the significance. *p* values of less than 0.05 were considered to be significant. Correlations between volatile and nonvolatile compounds were analyzed using the Pearson correlation coefficient. And Pearson's correlation coefficient calculated by package Himse and visualized by package *pheatmap* in R.

## 3 RESULTS AND DISCUSSION

Not all the strains that make up the *Lactobacillus,* microbiota of vegetables and fruits may guarantee the same performance during processing. Therefore, their selection is indispensable. The main criteria for selection and the corresponding characteristics are usually divided into three main categories: (a) protechnological; (b) sensory; and (c) nutritional (Di Cagno, Coda, Angelis, & Gobbetti, 2013). In the preliminary experiment, different sensory was observed among various FVFs (data not shown). To investigate the variation and influence of *Lactobacillus* on the flavor of FVFs, metabolomics‐based method was utilized to screen the discriminated compounds in FVFs, which is fermented by different *Lactobacillus*.

### 3.1 Effect of different *Lactobacillus* species on the flavor and taste compounds in fermented mixed vegetables and fruits

#### 3.1.1 Volatile compounds

**Figure 1:**
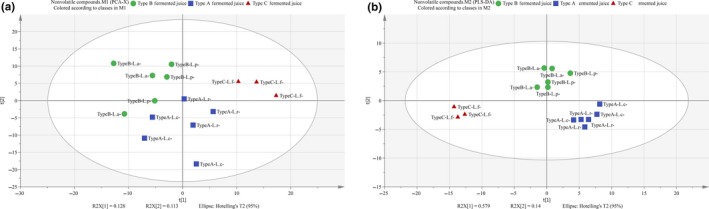
(A) PCA scores plot of the volatile compounds fragment ions of FVFs fermented by different *Lactobacillus* species. (B) PLS‐DA scores plot (R2Y = 0.953, R2X = 0.535, and Q2 = 0.917): (●) Type A FVFs, (■) Type B FVFs, and (▲) Type C FVFs; L. a: *Lactobacillus acidophilus*, L. p: *Lactobacillus plantarum*, L. c: *Lactobacillus casei*, L. f: *Lactobacillus fermentum*, L. r: *Lactobacillus rhamnosus*

Most of volatile compounds were contributed to the aromas of foods. To investigate the effect of *Lactobacillus* species on volatile compounds formation, 1,164 fragment ions were extracted from each sample by XCMS online analysis. After preprocessing, multivariate analysis was used to clear whether the difference existed among the volatile profiles of different FVFs. The PCA score plot showed that different FVFs were divided into three distinct types, suggesting that the different species significantly affect the volatile compounds profiles of FVFs (Figure 1A). It was partly explained why different sensory preference existed among various FVFs. Subsequently, a PLS‐DA model was constructed to discover the maker compounds that contributed to discrimination among different types of FVFs. The PLS‐DA model was validated by a sevenfold cross‐validation (*p* = 0.001): fitness (R2Y = 0.884, R2X = 0.344) and permutation result (*n* = 100; intercept of Q2 = −0.263), indicating that the model was regarded as predictable, stable, and reliable model. On basis of PLS‐DA (Figure 1B), different volatile compounds types of FVFs were also clearly discriminated. Then, the volatile compounds contributing to the discrimination were screened by statistically analyzing the intensities of all fragment ions from all groups of FVFs by ANOVA (<0.05) and calculating their VIP values (<1.0; Jumhawan et al., 2013). Compounds were chosen as potential markers, which were listed in Table 1.

**Table 1:** Identification of discriminant volatile compounds from PLS‐DA in different FVFs

Compounds name	Group	RT (min)	VIP
2,3‐Butanedione	Ketone	4.0802	4.29214
(E)‐2‐Hexenal	Alcohol	9.82876	6.8762
Acetic acid, hexyl ester	Ester	11.6959	3.27731
Formic acid, hexyl ester	Ester	13.8223	2.73747
Acetic acid	Acid	16.2241	3.29003
3,7‐dimethyl‐1,6‐Octadien‐3‐ol	Alcohol	18.8595	1.86479
3‐methyl‐Benzaldehyde	Aldehyde	20.9403	2.92149
Acetoin	Ketone	11.698	5.23835

Note

FVFs: fruit and vegetable juices; PLS‐DA: partial least squares discriminant analysis; VIP: variable importance in the projection.

#### 3.1.2 Nonvolatile compounds

**Figure 2:**
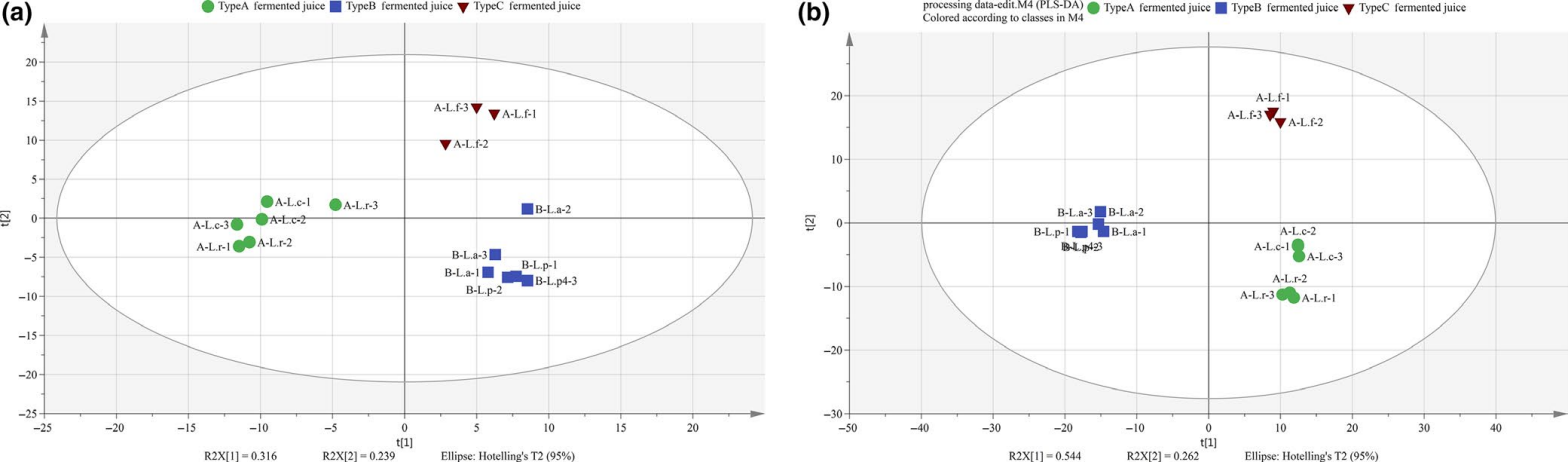
(A) PCA scores plot of the nonvolatile compounds fragment ions of FVFs fermented by different *Lactobacillus* species. (B) PLS‐DA scores plot (R2Y = 0.953, R2X = 0.535, and Q2 = 0.917): (●) Type A FVFs, (■) Type B FVFs, and (▲) Type C FVFs; L. a: *Lactobacillus acidophilus*, L. p: *Lactobacillus plantarum*, L. c: *Lactobacillus casei*, L. f: *Lactobacillus fermentum*, L. r: *Lactobacillus rhamnosus*

To investigate detailed information on the compounds responsible for the taste features of FVFs, nonvolatile compounds were detected and analyzed by a metabolomics approach. XCMS online was used to extract more than 4,000 ions from each sample. The fragment ions matrix was subjected to multivariate analysis after preprocessing. According to PCA score plot (Figure 2A ), FVFs fermented by different *Lactobacillus* species were also divided into three types, which is similar to the results of volatile compounds. Discriminant nonvolatile compounds were analyzed by PLS‐DA model (Figure 2B). Fourteen compounds were screened and listed in Table 2. The PLS‐DA model was validated by a sevenfold cross‐validation (*p* = 0.001): fitness (R2Y = 0.884, R2X = 0.344) and permutation result (*n* = 100; intercept of Q2 = −0.263), indicating that the model was regarded as predictable, stable, and reliable model. Three groups of compounds were screened, which included organic acid, amino acid, and carbohydrate. Most of these compounds contributed to the taste of fermented food. For example, L‐proline was widely considered to be compounds contributing to sweetness. The glutamic acid provided the umami taste in fermented foods (Ardö, 2006). Mannitol was an important taste compounds in fermented vegetable, which endowed fermented vegetable a fresh feeling (Jung et al., 2011).

**Table 2:** Identification of discriminant nonvolatile compounds from PLS‐DA in different FVFs

Compounds	Groups	RT (min)	VIP
Lactic acid	Acid	5.85624	5.90699
L‐proline	Amino	11.46	2.68632
Serine	Amino acid	13.2255	2.06464
Malic acid	Acid	16.4154	7.04869
L‐Aspartic acid	Amino	17.1432	1.17399
L‐Glutamic acid	Amino	19.4152	2.31066
Citric acid	Acid	23.9255	11.8969
D‐(‐)‐Fructose	Carbohydrate	25.4055	5.69528
D‐glucose	Carbohydrate	25.6952	11.5235
D‐mannitol	Carbohydrate	26.3965	7.78231

Note

FVFs: fruit and vegetable juices; PLS‐DA: partial least squares discriminant analysis; VIP: variable importance in the projection.

In general, diverse *Lactobacillus* species endowed FVFs significant different compounds profiles. Most of these compounds were contributor of FVFs' sensory. It explained that why different taste and aromas were obtained among different FVFs.

### 3.2 *Lactobacillus* plays different roles in formation of flavor and taste compounds in fermented mixed vegetables and fruits

To investigate the roles of *Lactobacillus* species on the vegetables and fruits fermented, changes of discriminate compounds were monitored during the fermentation (0, 6, 12, 24, and 48 hr). Fold changes of these compounds were calculated against those obtained from unfermented vegetable and fruits.

#### 3.2.1 Nonvolatile compounds

##### Amino

**Figure 3:**
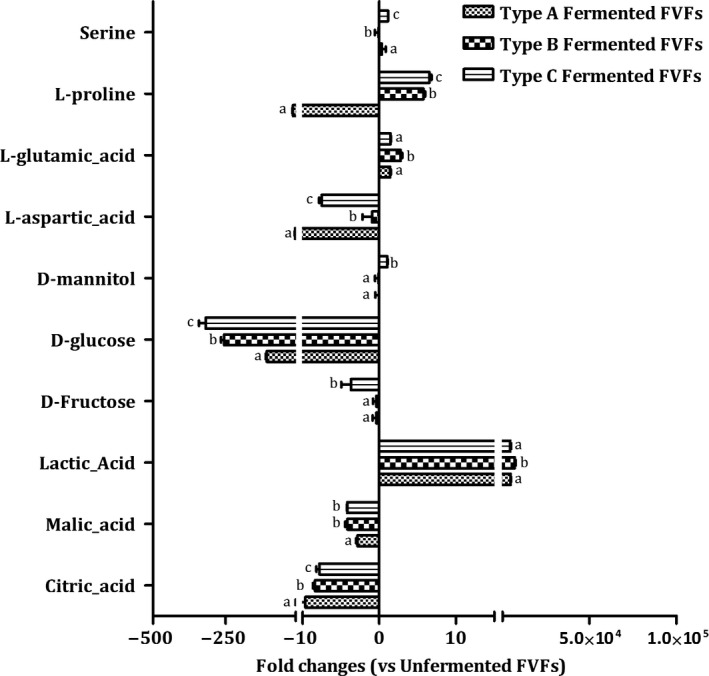
Fold changes of discriminant nonvolatile compounds

Four amino acids (Serine, L‐proline, L‐glutamic acid, and L‐aspartic acid) were screened as maker. Among these marker amino acids, L‐proline, L‐glutamic acid, and L‐aspartic acid were important taste compounds for food, which contribute to the taste of sweet and umami taste (Kato, Rhue, & Nishimura, 1989). The concentration of L‐glutamic acid showed up‐regulating against control (0 day) in all three types of FVFs, which mainly contribute to umami. Type A FVFs showed highest fold changes among three types of FVFs (Figure 3), indicating that *L. casei* and *L. acidophilus* might make a greater contribution to the umami taste for the FVFs. Furthermore, L‐aspartic acid, which is another contributor to the umami taste, decreased in all types of FVFs. In the previous studies, L‐aspartic acid was considered as an important precursors to produce volatile compounds such as acetoin and diacetyl (Ardö, 2006). Therefore, the reduction of L‐aspartic acid may contribute to producing the flavor compounds. Type C FVFs showed the highest reduction rate in the three types of FVFs. And *L. fermentum *may be an potential critical contributor to produce volatile compounds. In addition, the level of L‐proline, which has sweet taste, had the greatest change folds in type C FVFs, suggesting that *L. fermentum *may have some contributions to the sweet taste of FVFs.

##### Carbohydrate

Fructose, glucose, and mannitol were identified as marker for discriminating different types of FVFs (Figure 3). D‐glucose significantly decreased in every type of FVFs during the fermentation. The content of glucose showed highest change folds in type C FVFs, indicating that *L. fermentum* has better carbohydrate utilization of glucose in the blend fruit and vegetable juice. Interestingly, mannitol, which is a naturally occurring six‐carbon diabetic polyol that endow a refreshing and sweet taste for fermented vegetable, was only up‐regulating in type C FVFs (Jung et al., 2012), suggesting that *L. fermentum* may play an important role for the refreshing and sweet taste of FVFs. It is worth to note that mannitol was a metabolite produced from the fructose. In our results, fructose has the greatest changes in the type C FVFs. This result further indicated that *L. fermentum* played a critic role to produce the mannitol for endowing the refreshing and sweet taste to the FVFs.

##### Acid

The concentration of the metabolites such as citric acid and malic acid significantly decreased in every type of FVFs during the fermentation (Figure 3). Citrate is present in many of the substrates which are used for food fermentations such as fruits, vegetables, and milks. Its degradation usually results in the formation of unusual fermentation products such as diacetyl, acetoin, butanediol, and acetaldehyde (Hugenholtz, 1993). The highest change of citric acid and malic acid concentration was showed in type A FVFs, suggesting that *L. casei* and *L. rhamnosus* may be an important contributor for the volatile compounds producing. Conversely, the content of lactic acid, which is a main metabolites of lactic acid bacteria, showed up‐regulating against control (0 day) in all three types of FVFs. It mainly contributes to sour in fermented foods. Type B FVFs showed highest fold changes among three types of FVFs, indicating that *L. plantarum* and *L. acidophilus* might make a greater contribution to the sour taste for the FVFs.

#### 3.2.2 Volatile compounds

##### Esters

**Figure 4:**
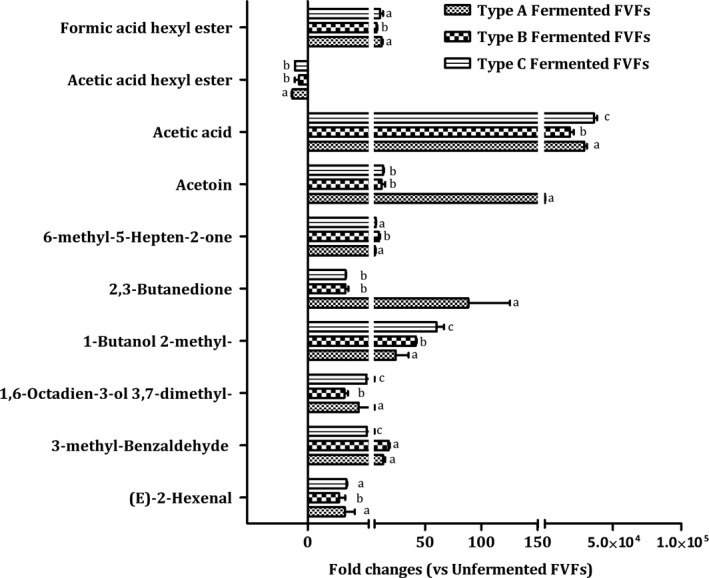
Fold changes of discriminant volatile compounds

Esters can influence the organoleptic characteristics well below their flavor threshold due to synergistic effects with other compounds, even if they are present at low concentrations (Dack, Black, Koutsidis, & Usher, 2017). Two esters were selected as volatile marker to discriminate different types of FVFs (Figure 4). A marked increase of these compounds was found in type A FVFs. Although formic acid hexyl ester and acetic acid hexyl ester naturally occurred in fruits, such as apple, mango, and banana, *Lactobacillus* affected the content of these compounds by indirect way, such as producing the acids to damage the cell of fruit, and promoting the release of these compounds (Zhao et al., 2007).

##### Acids

Moreover, a marked increase of acetic acid was found in every FVFs, especially in type C samples. Acetic acid was an important flavor compound imparting desirable sour flavor for various kinds of fermented foods, such as vinegar and pickles. This compound was mainly generated through heterolactic fermentation of glucose by *Lactobacillus*. The content of acetic acid has highest fold changes in type C FVFs due to inoculating heterofermentative lactic bacteria (*L. fermentum*). The results indicated that all the five species of *Lactobacillus* could produce acetic acid, and *L. fermentum* had better acetic acid producing capability than other species.

##### Ketone

Four ketones were identified as marker (Figure 4). Previous studies have demonstrated that acetoin and diacetyl (2,3‐butanedione) endowed desirable buttery flavor and odor to many fermented foods including cheese, which were generated through the metabolism of citrate by lactic acid bacteria. The levels of acetoin and diacetyl (2,3‐butanedione) were increased at the end of fermentation in all types of FVFs. And type A showed highest fold changes among all types of FVFs, indicating that *L. casei *and *L. rhamnosus* were the main contributor for these compounds during the FVFs fermentation and could endow FVFs desirable buttery flavor and odor. As previous studies have demonstrated that *L. casei* and *L. rhamnosus*, which were utilized in several fermented foods, produced diacetyl as one of important volatile constituents (Branen & Keenan, 1971; Jyoti, Suresh, & Venkatesh, 2003). 2‐Methylbutanol, which is a compound degradated from Ile, has the onion and fruity odor in the food (Ardö, 2006). Type C FVFs have the greatest change folds among various types of FVFs, suggesting that *L. fermentum* play an important role in producing 1‐butanol, 2‐methyl.

##### Aldehydes

The concentration of three aldehydes was identified as marker to differentiate various FVFs (Figure 4), which is naturally occurring in the fruits and vegetables. The levels of 3,7‐dimethyl‐1,6‐Octadien‐3‐ol, 3‐methyl‐benzaldehyde, and (E)‐2‐hexenal significantly increased in three types of FVFs during the fermentation. That is probably because that *Lactobacillus* affected the content of these compounds by indirect way, such as producing the acids to damage the cell of fruit, and promoting the release of these compounds (Zhao et al., 2007).

### 3.3 Metabolite correlation of FVFs during fermentation

**Figure 5:**
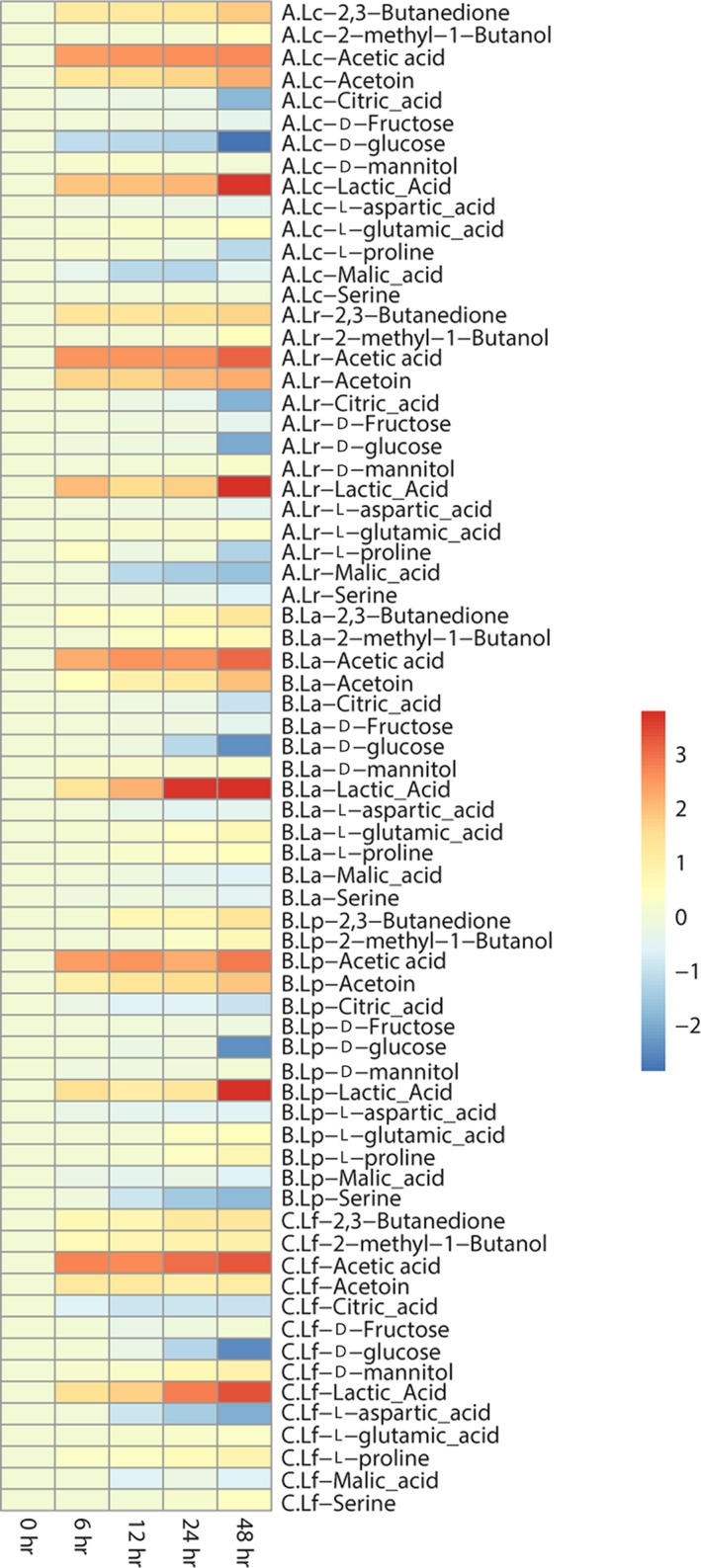
Changes of markers during the fermentation in different type of FVFs

To investigate the changes of these metabolites during FVFs fermentation, the contents of markers were monitored. According to the Figure 5, the content of lactic acid increased more significantly during fermentation in the FVFs inoculated with *L. plantarum*, *L. rhamnosus*, *L. casei,* and *L. acidophilus*, especially in the FVFs fermented by *L. plantarum*, which indicates that, homolactic fermentation has greater contribution to producing lactic acid. Homofermentative lactobacilli metabolize hexoses via the Embden‐Meyerhof pathway, yielding two moles of lactate and two moles of ATP per mole of glucose. Lactate is the sole or major product of homofermentative metabolism of carbohydrates in food (Gaenzle, 2015). Conversely, heterofermentative lactobacilli lack the gene coding for phosphofructokinase and normally metabolize hexoses via the phosphoketolase pathway, yielding lactate, acetate, ethanol, CO_2_, and only one mole of ATP per mole of glucose (Duar et al., 2017). An extra ATP can be gained by the production of acetate instead of ethanol. The FVFs fermented by *L. fermentum*, which is heterolactic fermentation, have a faster speed to accumulate acetic acid. Heterofermentative lactobacilli occur in most plant fermentations including wine, cider, cereal porridges and sourdough, and sauerkraut or kimchi. They cause an increase in acetate, which has antibacterial and antifungal activity, impacting flavor and sour taste. The redox potential and the antioxidant capacity of fermented foods are strongly influenced (Gaenzle, 2015). In addition, type A species inoculated to the blend fruit and vegetable juices more significantly increased the content of acetoin and 2,3‐butanedione during the fermentation, further indicating that type A species have greater contribution to these volatile compounds. Interestingly, the content of citric acid and L‐aspartic acid decreased more rapidly in the type A species, which are the precursors of acetoin and 2,3‐butanedione. Moreover, the content of mannitol and acetic acid raised more rapidly in the FVFs inoculated with *L. fermentum* than other species of *Lactobacillus*. It is worth to note that the fructose decreased more rapidly with increasing of mannitol in the FVFs inoculated with the *L. fermentum*. In the presence of fructose, the heterofermentative *Lactobacillus* is able to produce high levels of mannitol (Jumhawan et al., 2013; Wu et al., 2015). Mannitol is a maker compound for fermented vegetable and fruit and imparts a refreshing taste with noncarcinogenic properties. In the previous studies, the high level of mannitol produced by *Leuconostoc mesenteroides* was found in kimchi (Grobben et al., 2001). The regeneration of reducing equivalents can be achieved by reducing fructose to mannitol by the activity of an NADH‐linked dehydrogenase. *L. fermentum*, which is a species of heterofermentative lactic acid bacteria, plays an important role in generating these flavor compounds to modify the refresh and sweet taste of FVFs.

**Figure 6:**
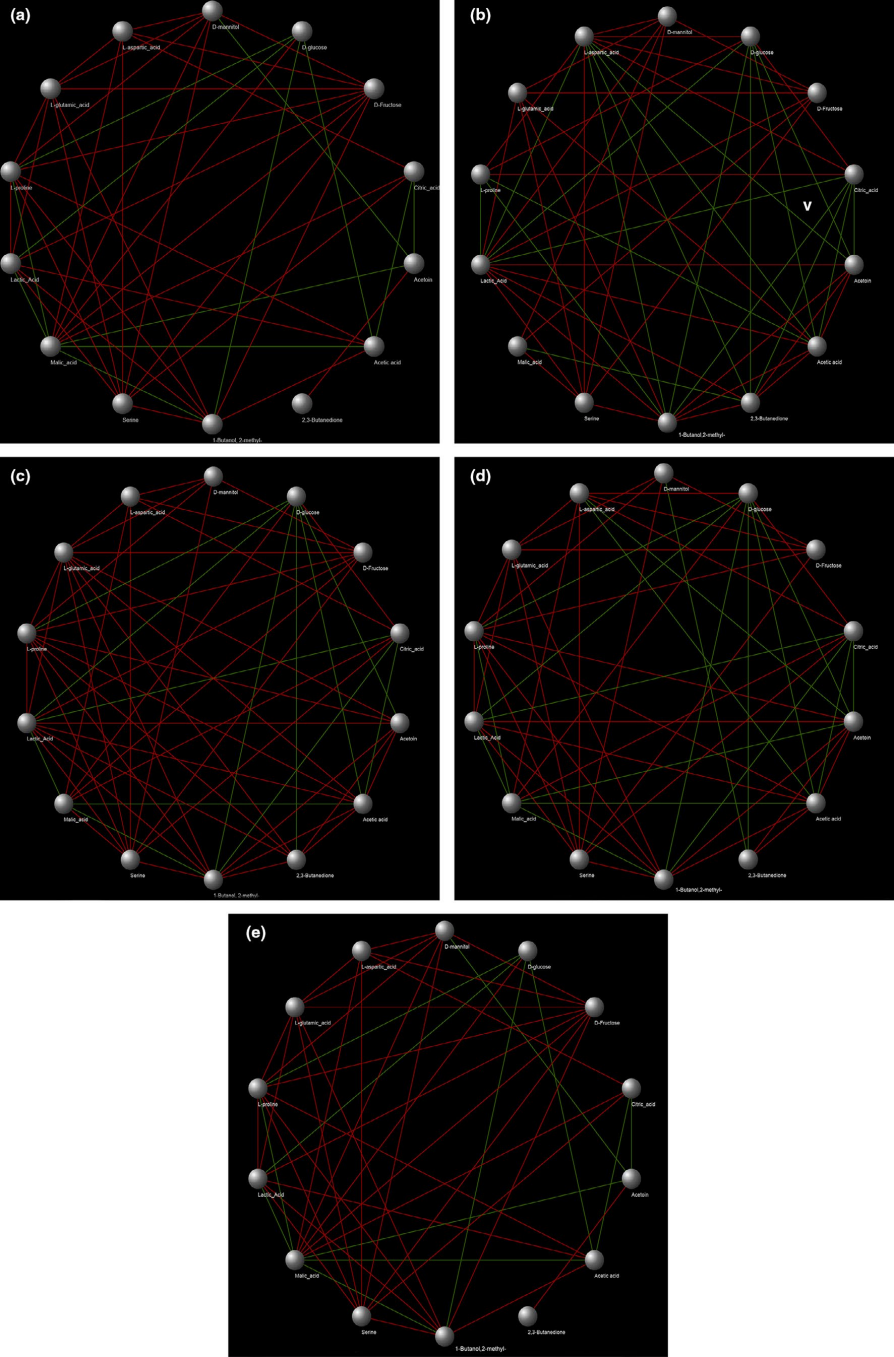
Correlation analyses among different markers by network (a. *Lactobacillus casei*; b. *Lactobacillus rhamnosus*; c. *L. rhamnosus*; d. *Lactobacillus plantarum*; and e. *Lactobacillus fermentum*)

To investigate the relationship between these markers, pairwise correlational comparisons were conducted between the 14 metabolites and network was constructed by the Cytoscape according to the spearson index (Figure 6). According to the Figure 6, a negative relationship was represented between citric acids and acetoin. It is worth to note that acetoin has significantly negative correlation with the content of citric acid and Asp in type A FVFs, but only citric acid or asp has significant negative correlation with acetoin in the type B or type C FVFs. Asp as a precursor substance could produce the α‐keto acid oxaloacetate catalyzed by the aminotransferase activity, and this compound may be catabolized into acetoin by *L. casei* (Thage et al., 2005). In addition, citric acid was another precursor substance to generate the acetoin. It might be explained that why the content was highest in the type A FVFs (Xiong, Li, Guan, Peng, & Xie, 2014). 2,3‐butanedione, providing sweet and butter flavor of fermented food, was positively correlated with the content of acetoin. In the previous studies, 2,3‐butanedione was a product generated from acetoin. Mannitol, contributing to the refreshing and sweet taste, was highest positive relation with fructose in the type C FVFs, which is mainly produced by heterofermentative lactic acid bacterium. Moreover, the content of acetic acid and lactic acid increased as decreasing of glucose. All the results suggested that different species of *Lactobacillus* play various roles due to the metabolic difference between different species of *Lactobacillus*.

In our studies, inoculating five different species of *Lactobacillus* obtained three different types of FVFs on basis of the flavor compounds. And 14 compounds were identified to discriminate different types of FVFs. Different species of *Lactobacillus *contribute to produce different flavor compounds. For example, *L. fermentum*, which is a species of heterofermentative lactic acid bacterium, plays an critical role in endowing FVFs to the sour and sweet taste due to producing acetic acid and mannitol. *L. casei* and *L. rhamnosus* have greatest contribution to produce the acetoin and 2,3‐butanedione, which provide butter flavor in fermented food. Christensen and Pederson (1958) reported that levels of acetoin increased only when inoculated homofermentative but not heterofermentative lactic acid bacteria to the medium with addition of citric acid. Overall, selection of mixed starter on the basis of the metabolic feature of bacteria is critical to modify the quality of FVFs.

## 4 CONCLUSION

In summary, this investigation addresses the effect of different species on the flavor compounds of FVFs by metabolomics analysis. Different *Lactobacillus* strains isolated from the fermented vegetable and fruit play different roles in modifying these compounds related to flavor features. These results may facilitate screening for better starters or mixed species starters to achieve better and certain quality characteristics.

## CONFLICT OF INTEREST

The auths have declared no conflict of interest.

## ETHICAL REVIEW

This study does not involve any humananimal testing.
